# A Comparison of Barbed Sutures and Standard Sutures with regard to Wound Cosmesis in Panniculectomy and Reduction Mammoplasty Patients

**DOI:** 10.1155/2016/7590396

**Published:** 2016-11-29

**Authors:** Kristen Aliano, Michael Trostler, Indira Michelle Fromm, Alexander Dagum, Sami Khan, Duc Bui

**Affiliations:** ^1^Department of Plastic Surgery, University of Texas Medical Branch, Galveston, TX, USA; ^2^Department of Plastic Surgery, Stony Brook University Hospital, Stony Brook, NY, USA

## Abstract

Cosmesis is a vital concern for patients undergoing plastic and reconstructive surgery. Many variations in wound closure are employed when attempting to minimize a surgical scar's appearance. Barbed sutures are one potential method of achieving improved wound cosmesis and are more common in recent years. To determine if barbed sutures differ from nonbarbed in wound cosmesis, we conducted a single-blinded, randomized, controlled trial of 18 patients undergoing bilateral reduction mammoplasty or panniculectomy. Patients were their own controls, receiving barbed sutures on one side and standard sutures on the contralateral side. Surgical scars were evaluated postoperatively by patient preference self-assessment and an observer. Ten patients were evaluated at 3 months postoperatively, yielding a mean Stony Brook Scar Evaluation Scale (SBSES) rating of 4.4 for barbed suture and 3.5 for regular suture (*p* = 0.15). At 6 months, 8 patients performed self-assessment to determine their preference; 4 preferred the barbed sutures, 1 preferred the regular sutures, and 3 had no preference. Further research with larger sample sizes is needed to determine if barbed sutures convey any advantage over standard sutures in wound healing. However, our results suggest that barbed sutures are a reasonable alternative to standard sutures particularly with regard to wound cosmesis.

## 1. Introduction

For patients who undergo plastic and reconstructive surgery, the appearance of surgical scars is an important consideration. Particularly, in the realm of aesthetic surgery, patients want scars that are easily camouflaged, have minimal hypertrophy and hyper- or hypopigmentation, and are not irregular in contour. Selecting an appropriate method of wound closure is consequently of paramount importance to surgeons, who seek to optimize postoperative scar appearance while minimizing surgical complications. To achieve these goals, surgeons employ a wide variety of options for wound closure, including topical adhesives, absorbable staples, and at least 5,269 types of sutures [1]. Barbed sutures present one such option, differing from most types of standard (smooth, nonbarbed) sutures in their ability to anchor tissues without the use of knots. Whereas the use of standard sutures is thought to increase risk of tissue ischemia due to increased tension at individual suture loops, the use of barbed sutures distributes tension across the length of a wound [2, 3]. By reducing mechanical stress on the skin, particularly at the wound edges, barbed sutures may potentially result in improved scar cosmesis. Prospective studies comparing the use of standard sutures and barbed sutures suggest that complication rates between the two are not significantly different or that the complications are lower in procedures involving barbed sutures [4–6]. Additionally, the use of barbed sutures may decrease operative times, in turn decreasing operative costs and increasing surgical efficiency.

Both unidirectional and bidirectional barbed sutures are available, including the unidirectional V-Loc™ (Covidien) and the bidirectional Quill™ (Angiotech). Bidirectional barbed sutures differ from unidirectional in that they have needles on both ends; the direction of the barbs changes at the midpoint of the suture [7]. Bidirectional barbed sutures have not been studied previously in panniculectomy patients, although there have been studies using them in abdominoplasty patients [8, 9]. The use of unidirectional barbed sutures in reduction mammoplasty patients has been previously reported in a study involving multiple types of procedures [5]. We performed a prospective matched-pairs study comparing postoperative scar cosmesis, complication profiles, and operative time for incisions closed with barbed sutures and incisions closed with standard sutures.

## 2. Methods

Over a period of 18 months, patients undergoing bilateral reduction mammoplasty or panniculectomy were recruited from the practice of two plastic surgeons. A total of 27 patients were initially enrolled in the study, all of whom were female, with 18 patients completing at least one follow-up visit with survey and scar evaluation. Panniculectomy was performed in 5 patients, with bilateral mammoplasty performed in the remaining 13 patients. Demographic data was collected and is shown in Table 1. Inclusion criteria were age >18 years old and patients undergoing panniculectomy or bilateral reduction mammoplasty. Exclusion criteria were age <18 years old, unilateral procedure, or no follow-up visits. To allow patients to act as their own controls, each reduction mammoplasty patient was randomized to have barbed sutures used on either the right or the left breast; standard sutures were used on the contralateral breast. Panniculectomy patients were randomized to have barbed sutures used on either the right or left half of the surgical incision; standard sutures were used on the contralateral side. Bidirectional Quill™ sutures were used for incisions closed with barbed sutures; monocryl or vicryl sutures were used for incisions closed with standard sutures. All patients were blinded as to which type of suture was used on which breast or incision half. Intraoperative details by type of suture, including incision length and time to achieve closure, were recorded. At one week postoperatively, patients were evaluated for complications. At 3-, 6-, and 12-month follow-up visits, patients rated their scars' overall appearances using a visual analog scale. At the time of these follow-ups, an observer also evaluated the patients' scars using the Stony Brook Scar Evaluation Scale (SBSES) which can be seen in Table 2 [10]. This observer was meant to be blinded to the side as well but had access to the patient charts and could potentially check which side had barbed and nonbarbed sutures. Statistical comparison of standard and barbed sutures was performed using dependent *t*-tests for paired samples, and ANOVA testing was used to compare across time points. This study was reviewed and approved by the IRB and informed consent was obtained from all subjects.

## 3. Results

The length of the incision closed by each type of suture was recorded for 11 patients with mean incision length not statistically different between groups: 26.6 cm for standard and 26 cm barbed sutures (*p* = 0.24). The amount of time to close each incision was recorded for 14 patients. Mean time to achieve closure significantly differed by type of suture used: 36.3 minutes for standard sutures and 24.4 minutes for barbed sutures (*p* = 0.003). Complications from the procedures and results of the follow-up evaluations are shown in Table 3.

At 1 week postoperatively, 11 patients' incision sites were evaluated for complications. One adverse event was recorded for incisions closed with each type of suture. One bilateral mammoplasty patient reported nipple numbness and bruising on the breast closed with barbed sutures, while another bilateral mammoplasty patient was found to have 3 cm vertical dehiscence of the breast closed with standard sutures.

At 3 months postoperatively, a nonblinded observer used SBSES to rate the appearance of 10 patients' scars on a scale of 0 to 5. Observer evaluation yielded a mean rating of 4.4 for barbed sutures and 3.5 for standard sutures, but this was not statistically significant (*p* = 0.15). Additionally, the patients performed a self-assessment of their scars, with 6 patients preferring side closed with barbed sutures, 1 patient slightly preferring the standard sutures, and 3 patients expressing no preference. The patient who slightly preferred the standard suture was also the patient who experienced nipple numbness in the side with barbed suture.

At 6 months postoperatively, a nonblinded observer again rated the appearance of 8 patients' scars, which was not statistically significant, with a mean rating of 3.75 for barbed sutures and 3.13 for standard sutures (*p* = 0.44). The patients self-assessed their scars, with 4 patients preferring the incision closed with barbed sutures, 1 patient with slight preference for the incision closed with standard sutures, and 3 patients expressing no preference. Two of the 3 patients with no current preference had no preference previously, and the 1 patient with slight preference for standard previously reported no preference.

At 12 months postoperatively, a nonblinded observer recorded a final rating for the appearance of 4 patients' scars, yielding a mean rating of 4.75 for barbed sutures and 4.25 for nonbarbed sutures (*p* = 0.39). On self-assessment 1 patient preferred the barbed sutures, 2 patients preferred standard sutures, and 1 patient expressed no preference. Only one of the patients in the 12-month group had follow-up at all time points and preferred the Quill suture at each point. One of the two patients who preferred standard suture was also the patient who had nipple numbness on the barbed suture side at the initial follow-up visit. The one patient who expressed no preference had previously expressed no preference.

When attempting to compare the SBSES across the time points for the barbed suture group, it was found on ANOVA test that there was no statistically significant difference between the 3-, 6-, and 12-month time points: 4.4, 3.75, and 4.75, respectively (*p* = 0.34). When comparing the nonbarbed suture groups with ANOVA testing at 3-, 6-, and 12-month time points, 3.5, 3.13, and 4.25, respectively, there was similarly no statistically significant difference (*p* = 0.5).

## 4. Discussion

A previous study of barbed suture closure of the Pfannenstiel incision in 188 patients suggests that scar cosmesis in wounds closed with Quill™ sutures is comparable to that of wounds closed with standard sutures [6]. Scar cosmesis in wounds closed with unidirectional barbed sutures is also comparable or superior to that of standard sutures in studies involving blinded evaluators [4, 11]. The results of our study similarly suggest that, at 3, 6, and 12 months postoperatively, there is no significant difference in mean cosmesis scores between wounds closed with barbed sutures and wounds closed with standard sutures. However, the possibility of scoring bias in our study cannot be discounted as the observer who assigned scar cosmesis scores was not blinded as to which type of suture was used on each side. Of additional note, at least 5 scar evaluation scales, including the scale used in this study, are available to clinicians/researchers, and it has been suggested that these scales are more suited to assess change in an individual than between individuals [12]. The existence of multiple scar scales also may complicate comparison of results between studies that use different scales.

To our knowledge, this is the first study of scar cosmesis in wounds closed with barbed sutures in which patients were asked to evaluate their own scars. At 3 months postoperatively, 10 patients evaluated their scars, of which 9 preferred the barbed suture closure or had no preference. Likewise, at 6 months postoperatively, 8 patients evaluated their scars, of which 7 preferred the barbed suture closure or had no preference. Patients did appear to show a slight preference for the standard suture closure at 12 months postoperatively; however, the small size with only 4 patients with follow-up records at this time lacks the power to make a definitive statement. Two of the four patients at the 12-month time point had not been previously evaluated. While our sample sizes at each stage are too small to be statistically significant or to adequately power the study, these results anecdotally suggest that patient satisfaction is no worse for barbed suture closure than for standard suture closure as scar maturation proceeds. It is also important to note that in contrast to the outside evaluator who assigned scar cosmesis scores, patients were blinded as to which sutures were used on which side, thereby minimizing bias.

Despite the shortcomings of a small sample size, complication profiles for both types of sutures used in this study appeared comparable. Two patients in this study experienced postoperative complications, including one instance of wound dehiscence in an incision closed with standard sutures and one instance of nipple numbness and ecchymosis in an incision closed with barbed sutures. Tissue ischemia may result in wounds closed with standard sutures, as the knots necessary for closure cause unequal distribution of tension. This is important to note as pressure-induced ischemia and necrosis are major contributors to wound dehiscence [6]; however, due to the small sample size of this study and the low overall complication rate, it is not possible to determine whether wound dehiscence was prevented by the use of barbed sutures. Conversely, several retrospective reviews have reported complication rates that were significantly higher among wounds closed with barbed sutures [13, 14]. This conflict in clinical opinion indicates that additional research with a larger patient base is necessary to discern whether complications from incisions closed with barbed sutures differ significantly from standard sutures.

An important consideration for any surgical procedure is the duration, as shorter procedure times may be more cost-effective due to decreased operating room fees, anesthesia charges, and time the patient spends under general anesthesia. In our study, the use of barbed sutures decreased the time to closure over that of standard sutures by 32.8% (24.4 minutes versus 36.3 minutes, *p* = 0.003). In a 2011 study, Jandali and colleagues reported that use of barbed sutures decreased the duration of unilateral breast reconstruction by 50 minutes, although there was no significant difference in the duration of bilateral breast reconstruction [13]. According to their cost analysis, despite the increased cost of barbed sutures over standard sutures, a 50-minute decrease in operative time would save a total of $7600 in operating room and anesthesia charges. Studies of abdominal closure in deep inferior epigastric perforator (DIEP) flap breast reconstruction and donor site quilting in latissimus dorsi flap breast reconstruction found no significant differences in operating times [15, 16]. Moreover, it is important to note that although our results show a significant difference in incision closure times, this difference may not correspond to shorter operative times overall. Additional research is necessary to determine whether the use of barbed sutures actually significantly decreases operative time and, consequently, operating costs.

Major limitations of this study are the small population size, the lack of blinding by the scar evaluator, and the incomplete follow-up by the patients for all time points and completion of the surveys. Future directions would include expanding the procedures done with barbed sutures and including a large population size to increase the power of the study.

## 5. Conclusion

It is advantageous for surgeons to have multiple options for wound closure, to allow for complex closure situations, to reduce the risk of postoperative complications, and to maximize patient satisfaction with the appearance of their surgical scars. The results of this study suggest that barbed sutures provide a reasonable alternative to standard sutures, with similar complication rates, shorter operative times, and comparable cosmetic outcomes. However, further investigation is needed to assess the validity of these results.

## Figures and Tables

**Table 1:** Patient demographics.

Demographics	
	*N* (%)

Age (years)	38 (range 20–59)
Gender	
Female	18 (100)
Procedures	
Panniculectomy	5 (27.8)
Reduction mammoplasty	10 (55.6)
Unknown/incomplete chart	3 (16.7)
Race	
White	11 (61.1)
Black/African American	2 (11.1)
Hispanic	1 (5.6)
Unknown/not recorded	4 (22.2)

**Table 2:** SBSES, Stony Brook Scar Evaluation Scale [10].

	Scar category	Points
Width	>2 mm	0
	≤2 mm	1
Height	Elevated/depressed in relation to surrounding skin	0
	Flat	1
Color	Darker than surrounding skin	0
	Same color or lighter than surrounding skin	1
Hatch marks/suture marks	Present	0
	Absent	1
Overall appearance	Poor	0
	Good	1

**Table 3:** Complications and patient evaluation (SBSES, Stony Brook Scar Evaluation Score).

	Barbed	Nonbarbed	*p* value
1 week	Nipple numbness and ecchymosis	3 cm vertical dehiscence	
Time to suture (minutes)	24.4	36.3	0.003
SES			
3 months	4.4	3.5	0.15
6 months	3.75	3.125	0.44
12 months	4.75	4.25	0.39
Total across time	4.23	3.5	*0.08*
Patient preference			0.19
3 months	6 (60)	1 (10)	
6 months	4 (50)	1 (12.5)	
12 months	1 (25)	2 (50)	
ANOVA testing between different time points	*p* = 0.34	*p* = 0.5	
